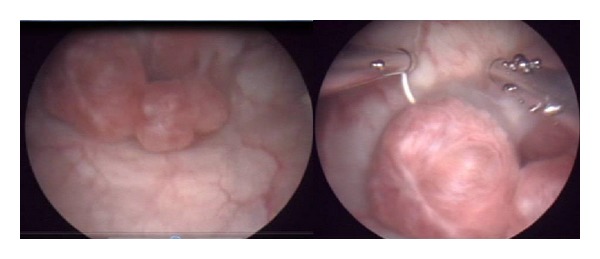# Bladder Endometriosis and Endocervicosis: Presentation of 2 Cases with Endoscopic Management and Review of Literature

**DOI:** 10.1155/2014/296908

**Published:** 2014-08-12

**Authors:** Javier Fuentes Pastor, Roberto Ballestero Diego, Miguel Ángel Correas Gómez, Eduardo Torres Díez, Alejandro Fernández Flórez, Gerardo Ballesteros Olmos, Jose Luis Gutierrez Baños

**Affiliations:** ^1^Service of Urology, Marqués de Valdecilla University Hospital, Faculty of Medicine, University of Cantabria, Santander, Spain; ^2^Department of Radiology, Marqués de Valdecilla University Hospital, Faculty of Medicine, University of Cantabria, Santander, Spain; ^3^Service of Gynecology and Obstetrics, Marqués de Valdecilla University Hospital, Faculty of Medicine, University of Cantabria, Santander, Spain

## Abstract

Urinary tract endometriosis and endocervicosis are an uncommon pathologic finding, with a common embryological origin. We present 2 cases of female patients with bladder mass. The first one was a finding of a nodular formation in the bladder during study of a nonviable foetus and the second was an incidental finding of a neoformation in the fundus of the bladder during the realization of an ultrasound. In both cases, we performed a surgical management with transurethral resection. Histopathological examination revealed a bladder endometrioma in the first case and endocervicosis with associated endometriosis in the second.

## 1. Introduction

Endometriosis is a chronic condition, usually gynaecological, of unknown cause. It is characterised by the presence of functionally active endometrial tissue (glandular epithelium and stroma) outside the uterine cavity, which induces a chronic inflammatory reaction. It is estimated to affect 5–10% of women of reproductive age, although in patients who present with pelvic pain the figures vary from 40 to 60% and among patients with sterility or infertility issues, the frequency of endometriosis reaches 20–40% [1].

The most common sites for external endometriosis are the ovaries, uterosacral ligaments, ovarian fossa, peritoneum of the Pouch of Douglas, and rectovaginal septum. It is less frequently found in the cervix, vagina, and vulva and is rare in the urinary (bladder and ureter), gastrointestinal, and respiratory tracts. Implants have been described in the skin and laparotomy scars, especially after Caesarean sections [2].

There is also a type of intramyometrial endometriosis known as adenomyosis, which can likewise be caused by Caesarean sections in hysterectomy scars. It is associated with an increased risk of placenta accreta and uterine rupture, and ectopic pregnancies have also been described in this location [3].

Bladder endometriosis is defined as the ectopic presence of endometrial tissue, glands, and/or stroma that invade the detrusor muscle and/or other planes of the bladder wall in an extrauterine site.

Involvement of the urinary tract is rare, occurring in only 1-2% of women with symptomatic endometriosis and affecting the bladder in most cases.

We present two cases, one of isolated endometriosis and the other of endocervicosis associated with endometriosis.

## 2. Case 1

The first case refers to a 38-year-old woman, ex-smoker, who had previously undergone 4 Caesarean sections but did not have any other urological history of interest.

**Figure 1:**
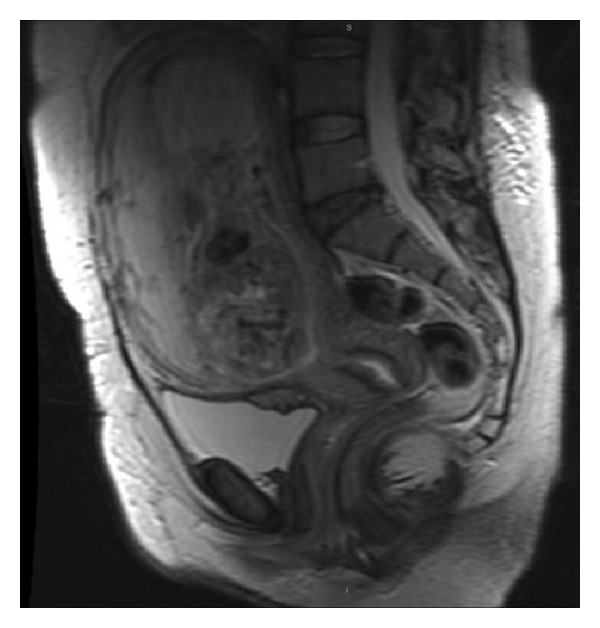


She was referred from the gynaecology department due to the finding of a nodular formation in the bladder during study of a nonviable foetus in week 22 of pregnancy. Prior to the Caesarean section, a magnetic resonance imaging (MRI) scan was performed, revealing an anterior placenta with signs of deep invasion of the myometrium related with placenta increta and a heterogeneous intensity and well defined nodular mass arising from the posterosuperior wall of the bladder of 7 × 30 × 18 mm suggestive of an endometrioma (Figure 1).

During the surgery (performance of the Caesarean section), placenta accreta and uterine rupture were discarded, and bladder integrity was confirmed by palpation of an indurated nodule that increased the thickness of the wall in the centre of the vesicouterine angle.

**Figure 2:**
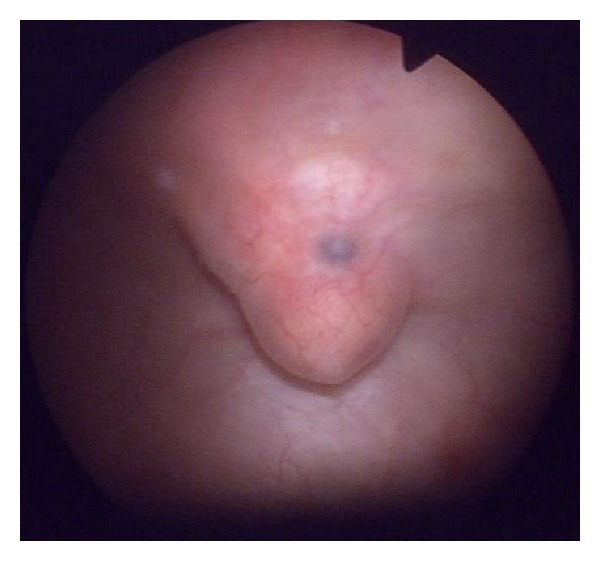


Cystoscopy was performed, in which a raised nodular formation was observed in the fundus of the bladder, bluish inside surrounded by a yellowish area (Figure 2).

In view of these findings, complete transurethral resection of the lesion was carried out.

Histopathological examination revealed a bladder endometrioma.

The patient presently remains asymptomatic from a urological perspective, with no recurrence after 16 months of follow-up.

## 3. Case 2

The following case was a 64-year-old woman with a history of hypertension, insulin-dependent diabetes mellitus, dyslipidemia, primary hypothyroidism, and chronic renal failure. She had previously undergone 2 Caesarean sections.

**Figure 3:**
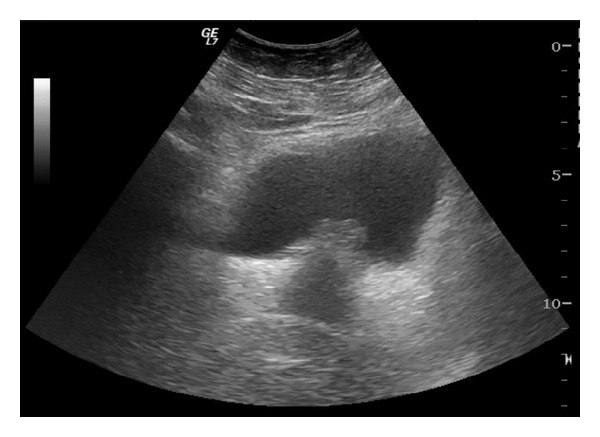


While performing renal Doppler ultrasound (Figure 3), a 2 cm filling defect was incidentally observed in the fundus of the bladder, consistent with a neoformation. Although the patient was asymptomatic from a urological point of view, transurethral resection of the lesion was performed.

During the resection, a solid, adenomatous mass was observed, around 2 cm in diameter with a pedunculated base, which was completely excised (Figure 4).

Histopathological study of the specimen revealed endocervicosis with associated endometriosis.

The patient remains asymptomatic from a urological point of view after 7 months of follow-up and is currently being monitored by the gynaecology department.

## 4. Discussion

Bladder endometriosis is characterised by the presence of endometrial tissue in the bladder detrusor muscle [4].

It is a tissue of Müllerian origin that can present in three forms: as endometriosis, endosalpingiosis, and endocervicosis, although endometriosis is the most common form [5].

The various forms can present alone or associated with one another; the presence of two or more of these lesions simultaneously is known as* Müllerianosis* [5].

It is estimated that urinary tract involvement in women with endometriosis is between 1 and 5% [6], with the bladder most often affected [7].

This entity was first described by Judd in 1921 [8]; later, in 1992, Clement and Young [9] described endocervicosis as a variant of endometriosis.

It can present as a primary lesion in women who have not previously undergone gynaecological surgery or may be secondary to pelvic surgery, essentially after a Caesarean section [5, 10].

Its aetiology is unclear, although there are several theories that attempt to explain its origin: the embryonic (polyvalent embryonic remnants), metaplastic, and immunological theories [10].

1. The implantation or retrograde menstruation theory: postulated by Sampson, endometriosis is the result of the implantation and growth in the peritoneum and ovary of fragments of endometrial tissue that migrate to the abdominal cavity during menstruation through the Fallopian tubes. Retrograde menstruation is observed in 90% of women, which suggests that additional factors are required: genetic, immunological, hormonal, and environmental factors that cause susceptibility to endometriosis.
2. The induction theory: it is not the endometrium itself that migrates but some substances released by it, which cause differentiation from mesenchymal cells present in the abdominal connective tissue.
3. *In situ* development theory: according to this theory, the ectopic endometrium develops “*in situ*” from local tissues, including the germinal epithelium of the ovary and remnants of the Wolff and Müller ducts and from pluripotent cells present in the peritoneal serosa. This is the theory that best explains the disease for cases of atypical or prepubertal sites.

However, the migratory theory by retrograde menstruation through the Fallopian tubes, post-pelvic-surgery seeding, and blood or lymphatic seeding is the most widely accepted hypothesis, as occurs in our two cases in which both patients had a previous surgical history of Caesarean sections [5, 10].

Clinical manifestations are usually nonspecific and may include pelvic pain, dysuria, tenesmus, pollakisuria, and even acute urinary retention. Haematuria may also appear, typically during menstruation, although it occurs in only 20–35% of patients. Symptoms are often exacerbated at this time [7, 11, 12].

Urine cultures and cytology studies are generally negative. Filling defects in the bladder wall can usually be observed on ultrasound and other imaging tests [10, 13].

Transvaginal ultrasound appears to have better diagnostic accuracy than transabdominal ultrasound for evaluating the extension towards the uterus and vesicovaginal septum. MRI allows better identification of the lesion and assessment of bladder wall infiltration [13].

Cystoscopy enables a diagnosis to be established in 65–72% of patients, although the definitive diagnosis is histopathological. Dark blue solid lesions are usually observed, with an oedematous halo with bullae or cysts. The characteristics of these lesions generally vary throughout the menstrual cycle, both in size and in pigmentation [5, 7, 14].

Transurethral resection is diagnostic and therapeutic and allows samples to be obtained for histopathological analysis. The definitive diagnosis is established by the presence of endometrial glands in the bladder wall [15]. In the case of endocervicosis, uterine cervix glands can be observed [12].

There is no consensus as regards treatment of these lesions, although their final treatment is primarily surgical [5, 10, 15]. In the case of bladder endometriosis, hormone treatment may be commenced immediately with oral contraceptives, progestogens, or danazol [10, 11].

The choice of treatment depends on different factors, such as the size of the lesion, its location, or the number of tumours.

Many surgical modalities have been used for the treatment of these lesions: transurethral resection and both open and laparoscopic partial cystectomy [7]. Endoscopic resection of the lesions is currently considered the treatment of choice when possible [5, 7, 10].

As it is a rare entity, its evolution and prognosis have been evaluated in small case series, with clinical improvement in most patients [16]. There may be recurrence in up to 56% of cases of pelvic endometriosis [17]. In case of persistence or recurrence of symptoms, segmental bladder resection is indicated [17]. With respect to endocervicosis, no cases of recurrence have been described, despite follow-ups of up to 14 years [9].

## 5. Conclusion

Both bladder endometriosis and endocervicosis are rare entities with a common embryological origin. It is important to be aware of them in the differential diagnosis of benign and malignant bladder disease. Their clinical presentation may help in the suspected diagnosis of the entity. The treatment of choice is complete transurethral surgical resection, provided that the size and characteristics of the lesion allow.

## Figures and Tables

**Figure 4:**